# Asiatic Acid Inhibits Liver Fibrosis by Blocking TGF-beta/Smad Signaling *In Vivo* and *In Vitro*

**DOI:** 10.1371/journal.pone.0031350

**Published:** 2012-02-07

**Authors:** Li-xia Tang, Rui-hua He, Guang Yang, Jia-ju Tan, Li Zhou, Xiao-ming Meng, Xiao Ru Huang, Hui Yao Lan

**Affiliations:** 1 Institute of Medical Research, The First People's Hospital of Foshan, Foshan, Guangdong, China; 2 Department of Medicine, West China Hospital of Sichuan University, Chengdu, China; 3 Li Ka Shing Institute of Health Sciences and Department of Medicine and Therapeutics, The Chinese University of Hong Kong, Hong Kong, China; The University of Hong Kong, Hong Kong

## Abstract

Liver fibrosis is a major cause of liver failure, but treatment remains ineffective. In the present study, we investigated the mechanisms and anti-hepatofibrotic activities of asiatic acid (AA) in a rat model of liver fibrosis induced by carbon tetrachloride (CCl_4_) and *in vitro* in TGF-beta1-stimulated rat hepatic stellate cell line (HSC-T6). Treatment with AA significantly attenuated CCl_4_-induced liver fibrosis and functional impairment in a dosage-dependent manner, including blockade of the activation of HSC as determined by inhibiting *de novo* alpha smooth muscle actin (a-SMA) and collagen matrix expression, and an increase in ALT and AST (all p<0.01). The hepatoprotective effects of AA on fibrosis were associated with upregulation of hepatic Smad7, an inhibitor of TGF-beta signaling, thereby blocking upregulation of TGF-beta1 and CTGF and the activation of TGF-beta/Smad signaling. The anti-fibrosis activity and mechanisms of AA were further detected *in vitro* in HSC-T6. Addition of AA significantly induced Smad7 expression by HSC-T6 cells, thereby inhibiting TGF-beta1-induced Smad2/3 activation, myofibroblast transformation, and collagen matrix expression in a dosage-dependent manner. In contrast, knockdown of Smad7 in HSC-T6 cells prevented AA-induced inhibition of HSC-T6 cell activation and fibrosis in response to TGF-beta1, revealing an essential role for Smad7 in AA-induced anti-fibrotic activities during liver fibrosis *in vivo* and *in vitro*. In conclusion, AA may be a novel therapeutic agent for liver fibrosis. Induction of Smad7-dependent inhibition of TGF-beta/Smad-mediated fibrogenesis may be a central mechanism by which AA protects liver from injury.

## Introduction

Liver fibrosis represents the final common pathway of virtually all chronic liver diseases. It is characterized by the excessive accumulation of extracellular matrix (ECM) and activated hepatic stellate cells (HSC) that are undergoing myofibroblast transition identified by *de novo* a-SMA expression [1], [2]. Although a significant progress has been made in our understanding of hepatic fibrosis, treatment for liver fibrosis remains ineffective. Therefore, there is an urgent need for searching and developing antifibrotic strategies which can prevent, halt or reverse hepatic fibrosis.

In hepatic fibrosis, the excessive ECM, including collagen type I and III, is produced by activated mesenchymal cells which resemble myofibroblasts derived from quiescent HSC, periportal or perivenular fibroblasts, circulating fibrocytes, and bone marrow cells [1], [2]. Increasing evidence shows that TGF-beta1 is a key mediator in the process of liver fibrosis [3], [4]. The finding of increased HSCl activation and liver fibrosis in mice with tetracycline-regulated TGF-beta1 expression in the liver provides a direct evidence for a critical role of TGF-beta1 in hepatic fibrosis [5]. It is now clear that after binding to its receptors, TGF-beta1 activates its downstream signaling pathway, Smad 2 and Smad3, to mediate fibrosis, which is negatively regulated by Smad7, an inhibitor of TGF-beta signaling, via the ubiquitin-proteasome degradation mechanism [6], [7]. In the context of liver fibrosis, Smad3 is pathogenic because mice null for Smad3 are protected against dimethylnitrosamine-induced hepatic fibrosis [8]. In contrast, Smad7 is protective since deletion of Smad7 promotes, but overexpression of Smad7 protects against HSC activation and hepatic fibrosis in vitro and in vivo [9]–[11]. The inhibitory role of Smad7 in fibrosis is also found in chronic kidney disease [12]. We detected that disruption of Smad7 gene promotes renal fibrosis in a mouse model of obstructive nephropathy [13]. In contrast, overexpression of Smad7 is capable of inhibiting TGF-beta1 and angiotensin II-induced fibrosis in vitro [14], [15] and in a number of disease models including diabetic nephropathy [16]–[18]. However, it is also known that TGF-beta1 is an anti-inflammatory cytokine. Thus, therapies with general blockade of TGF-beta1 may risk in enhancing the inflammatory response, which has largely limited the development of anti-TGF-beta therapy clinically. Nevertheless, the better understanding of the mechanisms of TGF-beta/Smad signaling in diseases associated with fibrosis may be a critical step towards the development of novel and specific anti-fibrosis drugs.

Asiatic acid (AA) is one of the triterpenoid components found in *Centella asiatica* [19]. Many studies have shown that AA has a variety of pharmacological effects on anti-inflammation [20], [21], antioxidation [22], anti-tumor [23], [24], neuroprotection [25], [26], and wound healing [27], [28]. In particular, AA has been shown to be a hepatoprotective agent. A number of studies demonstrated that AA can protect liver from injury via mechanisms underlying anti-mitochondrial stress and cellular antioxidant system in cultured hepatocytes and Kupffer cells, and in a mouse model induced by D-galactosamine and lipopolysaccharides [29]–[31]. It has been also reported that AA is capable of inhibiting collagen matrix production by HSC and keloid fibroblasts by blocking the autocrine effect of TGF-beta1 *in vitro* [32], [33], however, the role and mechanisms by which AA inhibits liver fibrosis remain largely unknown. Therefore, the present study investigated the therapeutic effect and mechanisms of AA in a rat model of CCl_4_-induced liver fibrosis and *in vitro* in TGF-beta1-stimulated rat HSC-T6 cell line.

## Methods

### Asiatic Acid

Purified nature product of AA (95%) was obtained from Changzhou Natural Product Inc (Guangxi, China) and was used for in vivo treatment as described below, while the HPLC-purified AA (Sigma-Aldrich, St. Louis, MO) was used for *in vitro* studies.

### Animal Model of CCl_4_-Induced Liver Fibrosis and Asiatic Acid Treatment

Male Sprague-Dawley (SD) rats (6–8 weeks of age, 180–200 g) were obtained from the Guangdong Medical Laboratory Animal Center, fed with a standard laboratory diet and tap water in a temperature- and humidity-controlled animal house under 12-h light–dark cycles. Forty rats were divided randomly into five groups (n = 8 for each group) including: 1) normal control, 2) disease control, and 3) three AA treatment groups at doses of 0.5 mg/kg, 2 mg/kg, and 8 mg/kg, respectively. In addition, one group of normal 6 rats was treated with a dose of 8 mg/kg of AA as AA toxicity control. Except the normal control groups, all animals were treated with intra-peritoneal injection of 2 ml/kg of CCl_4_ (diluted in 20% peanut oil) twice per week for 6 weeks to induce liver fibrosis. For those received the AA treatment, animals were given with three different doses of AA (0.5, 2, and 8 mg/kg) suspended in a 1.5% methyl cellulose (MC) mixture by oral gavage daily for the 6 week-period, while rats from disease control group were treated with equivalent volumes of the MC mixture solution without AA. Normal control animals were also received the same volumes of peanut oil equivalent to the CCl_4_-treated animals. To exclude the toxicity of AA *in vivo*, one group of normal 6 rats was treated with a dose of 8 mg/kg of AA following the same experimental protocol of AA treatment. At the end of the sixth week, all of rats were sacrificed under anesthesia with 3% sodium pentobarbital (45 mg/kg, ip). Blood samples and liver specimens were obtained for analyses of liver functions, mRNA and protein expression of fibrotic indexes by real-time reverse transcription polymerase chain reaction (RT-PCR), Western blot, histology, and immunohistochemistry. All experimental procedures were approved by the Animal Experimental Committee at the First People's Hospital of Foshan (A0331).

### Liver Function Test

Serum alanine transaminase (ALT) and aspartate transaminase (AST) activities, markers for hepatotoxicity, were detected with an automatic analyzer (Olympus, Japan) at the Department of Chemical Pathology.

### Histopathology and Immunohistochemistry

Changes in liver morphology were examined in methyl Carnoy's fixed, paraffin-embedded tissue sections (3 micrometer) stained with hematoxylin and eosin. The histopathological scores of fibrosis were evaluated following the published criteria [34]: 0) normal liver; 1) an increase in collagen matrix accumulation without the formation of septa (small stellate expansions of the portal fields); 2) formation of incomplete septa from the portal tract to the central vein (septa that do not interconnect with each other); 3) complete but thin septa interconnecting with each other to divide the parenchyma into separate fragments; 4) same as grade 3, except for the presence of thick septa (complete cirrhosis).

Immunohistochemistry was performed in paraffin sections using the microwaved-based antigen retrieval method as described previously [35]. Antibodies used in this study included: rabbit polyclonal antibodies to collagen I, III (Southern Tech, Birmingham, AL), and a-SMA (Sigma, St. Louis, MO). An irrelevant isotype rabbit IgG was used as a negative control. The stained sections were developed with diaminobenzidine to produce brown products and counterstained with hematoxylin.

Quantitation of immunostaining was carried out on coded slides as previously described [13], [18]. Expression of collagen I, III, and a-SMA in the liver cross-sections was determined using the quantitative Image Analysis System (AxioVision 4, Carl Zeiss, Jena, Germany). Briefly, 10 fields (×20) were randomly selected from each section and positive signals within the section were highlighted, measured, and expressed as percent positive area of the entire liver tissues examined.

### Cell culture

The HSC-T6 cell line was gifted by Professor SL Friedman (Liver Disease Research Center of San Francisco General Hospital, CA, USA). HSC-T6 cells were routinely cultured in DMEM (Gibco, USA) supplemented with 10% heat-inactivated fetal bovine serum (FBS).

We first determined the safe dosages of AA for the study, HSC-T6 cells were cultured at a density of 5×10^4^ cells/mL in 100 uL DMEM containing 0.2% FBS in 96-well microplates and AA or DMSO as control in various dosages (0.0, 2.5, 5.0, 10.0, 20.0, 30.0, 40.0, 60.0, and 80.0 micro molar) was added to the culture for 24 h. A dosage-dependent cytotoxicity of AA was measured by 3-(4, 5-dimethylthiazol-2-yl)-2, 5-diphenyl tetrazolium bromide (MTT) assay kit (Sigma-Aldrich) and lactate dehydrogenase (LDH) release kit (Sigma-Aldrich) following the manufacturer instructions. The extent of cytotoxicity and the IC_50_ of AA were calculated using the results of both MTT and LDH assays.

To determine the optimal dose of TGF-beta1 on collagen matrix expression, HSC-T6 cells were treated with TGF-beta1 (R&D System) at dosages of 0.0, 0.1, 0.5, 1.0, 2.0, and 5.0 ng/ml for various time of 0, 1, 3, 6, 12, 24 h. TGF-beta1-induced collagen I and III expression was determined at the mRNA level by real-time PCR and at the protein level by Western blot analysis.

To investigate the inhibitory effect and mechanism of AA on TGF-beta1-mediated fibrosis, HSC-T6 cells were pre-treated with AA at dosages of 0, 5, 10, 20, 30 micro molar for over night, followed by addition of an optimal dose of TGF-beta1 (1 ng/ml) for various time periods for examination of phospho-Smad2/3 and expression of Smad7, collagen I, and a-SMA by real-time and Western blot analysis as described below.

To confirm the protective role of AA in TGF-beta1-induced fibrosis in HSC-T6 cells *via* induction of Smad7, a stable HSC-T6 cell line with Smad7 knockdown was established. In brief, Smad7 siRNA was cloned into the P-super1 plasmid and transfected into HSC-T6 cells with lipofectamine 2000 following the manufacturer's protocol (Invitrogen). The cells were then selected with G418 in 100 mg/ml for one month and maintained in 50 mg/ml of G418. A stable HSC-T6 cell line transfected with P-super1 empty plasmid only was used as control.

### Real-time Reverse Transcription Polymerase Chain Reaction (RT-PCR)

Total RNA was extracted from frozen liver samples or cultured cells using the RNeasy Mini Kit (Qiagen Inc)following the manufacturer's protocol. mRNA expression of collagen I, collagen III, a-SMA, TGF-beta1, CTGF, Smad7, and GAPDH was detected by quantitative real time PCR using an Opticon 2 DNA Engine Real Time PCR Detection (MJ Research Inc., Waltham, MA) as previously described [13], [16], [17]. The expression levels of all the transcripts were normalized to that of the housekeeping gene GAPDH in the same tissue.

### Western Blot Analysis

Proteins extracted from either liver tissues or cultured HSC were analyzed by Western blotting as previously described [13], [16], [17]. Antibodies used in this study included: collagen I, collagen III (Southern Biotech), α-SMA(Sigma, St. Louis, MO), Smad7, phospho-Smad2/3 (Santa Cruz Biotechnology Inc., Santa Cruz, CA), GAPDH (Chemicon Inc., Temecula, CA), and IRDyeTM800 conjugated secondary antibodies (Rockland Immunochemical Inc., Gilbertsville, PA). Signals were scanned and visualized by Odyssey Infrared Imaging System (LiCor Inc., Lincoln, NE). The ratio of the protein interested was subjected to GAPDH and was densitometrically analyzed by Image J software (NIH, Bethsda, MD).

### Statistical Analyses

All data are expressed as mean ± SEM. The differences between multiple groups were evaluated by a one-way analysis of variances (ANOVA), followed by Newman-Keuls Post Test using Prism 4.0 Program (GraphPad Software, Inc. San Diego, CA).

## Results

### Asiatic Acid Treatment Inhibits CCl_4_-Induced Liver Functional and Histological Damage

**Figure 1:**
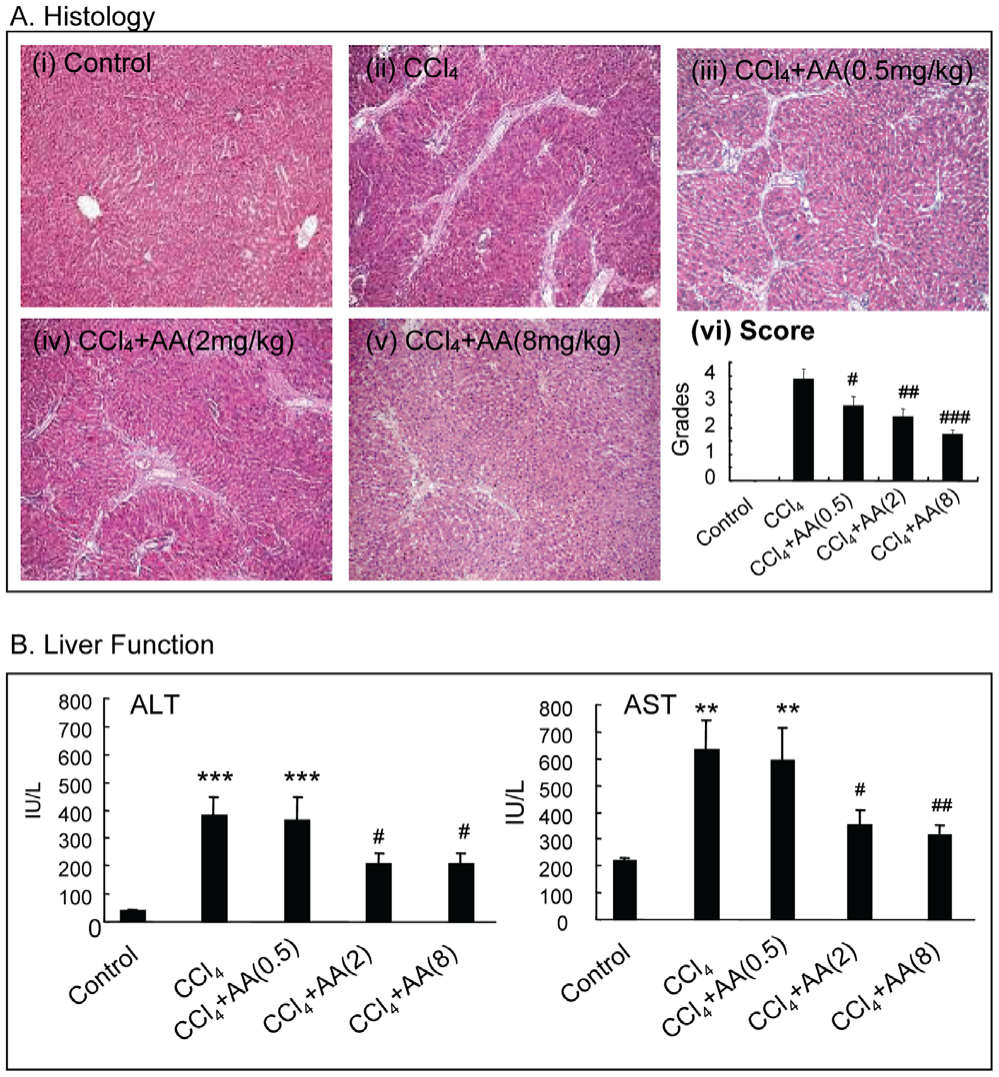
AA treatment attenuates CCl_4_-induced liver damage and functional impairment in a dosage-dependent manner in rats. **A**. Histology (H&E). **B.** Liver function. Data represent mean ± SEM for groups of 8 animals. **p<0.01, *** p<0.001 versus normal control; ^#^p<0.05, ^##^p<0.01, ^###^p<0.001 versus CCl_4_-induced disease control. Magnification: ×100.

Administration of CCl_4_ for 6 weeks caused a moderate to severe liver injury as demonstrated by the development of severe liver damage with thick fibrotic septa and pseudolobular formation (Fig. 1Aii, vi). Serologically, levels of ALT and AST were also highly significantly elevated in disease control rats when compared to normal control rats (Fig. 1B). In contrast, treatment with AA resulted in attenuation of both histological and functional injury in a dosage-dependent manner, being significant at doses of 2 and 8 mg/kg (Fig. 1A iii–vi and B). Normal rats treated with AA (8 mg/kg) exhibited normal histological and serological changes similar to the normal control rats (data not shown).

### Asiatic Acid Treatment Attenuates CCl_4_-Induced Liver Fibrosis *in vivo*

**Figure 2:**
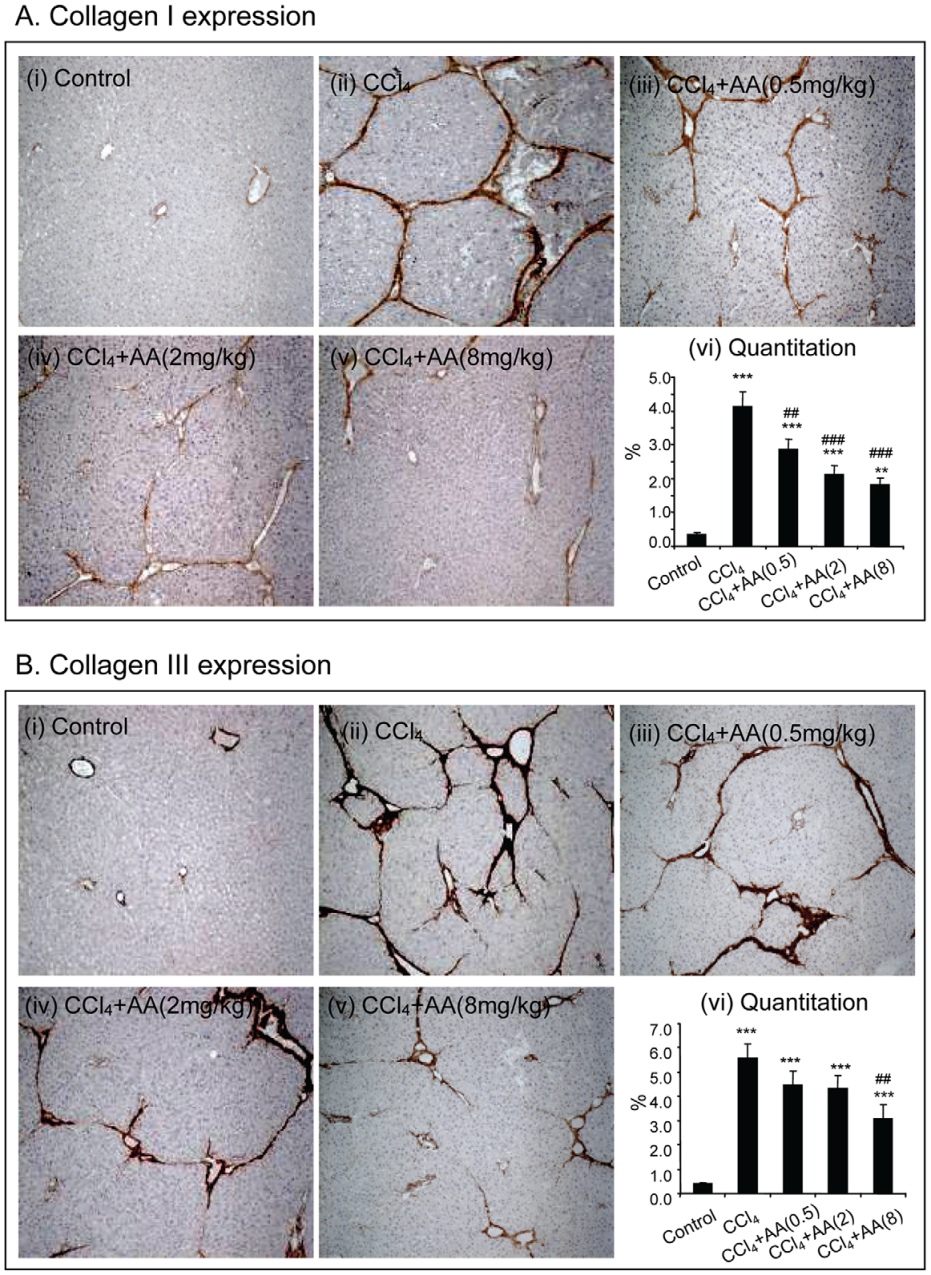
Immunohistochemistry detects that AA treatment attenuates CCl_4_-induced liver fibrosis in a dosage-dependent manner in rats. **A.** Collagen I expression. **B.** Collagen III expression. Data represent mean ± SEM for groups of 8 animals. **p<0.01, *** p<0.001 versus normal control; ^##^p<0.01, ^###^p<0.001 versus CCl_4_-induced disease control. Magnification: ×100.

**Figure 3:**
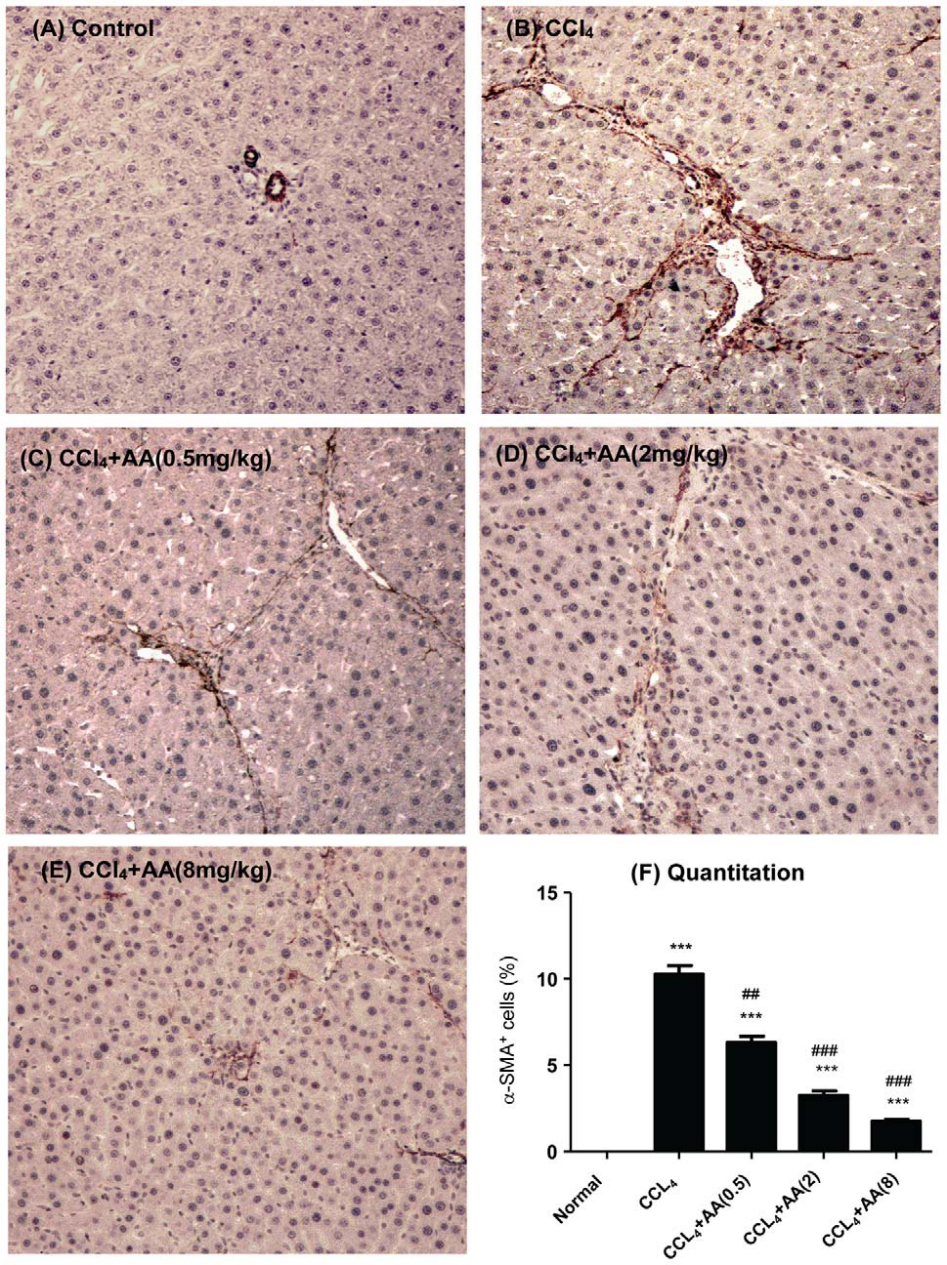
Immunohistochemistry detects that AA treatment attenuates CCl_4_-induced activation of HSC in a dosage-dependent manner in rats. Activation of HSC was determined by a-SMA+ myofibroblast transition. **A.** Representative picture from a control rat treated with 20% peanut oil. **B.** Representative picture from a rat treated with CCl_4_. **C–E.** Representative pictures from CCl_4_-treated rats received AA in a dosage-dependent manner. **F**. Quantitation of a-SMA+ cells. Data represent mean ± SEM for groups of 8 animals. *** p<0.001 versus normal control; ^##^p<0.01, ^###^p<0.001 versus CCl_4_-induced disease rats. Magnification: ×200.

We next examined the therapeutic effect of AA on liver fibrosis. As shown in Figure 2 , immunohistochemistry detected that compared to normal control rats, CCl_4_-treatment caused a remarkable collagen I and III accumulation in the liver (Fig. 2A and B, i–ii). In contrast, treatment with AA reduced hepatic collagen matrix accumulation in a dosage-dependent manner (Fig. 2 A and B, iii–v), which was confirmed by quantitative analysis (Fig. 2A and B, vi). Importantly, treatment with AA on protection of liver from CCl_4_-induced fibrosis was associated with inhibition of HSC activation as determined by blockade of a-SMA+ myofibroblast transition. As shown in Figure 3, addition of AA was capable of blocking a-SMA+ cell accumulation along the fibrotic septa in a dosage-dependent manner.

**Figure 4:**
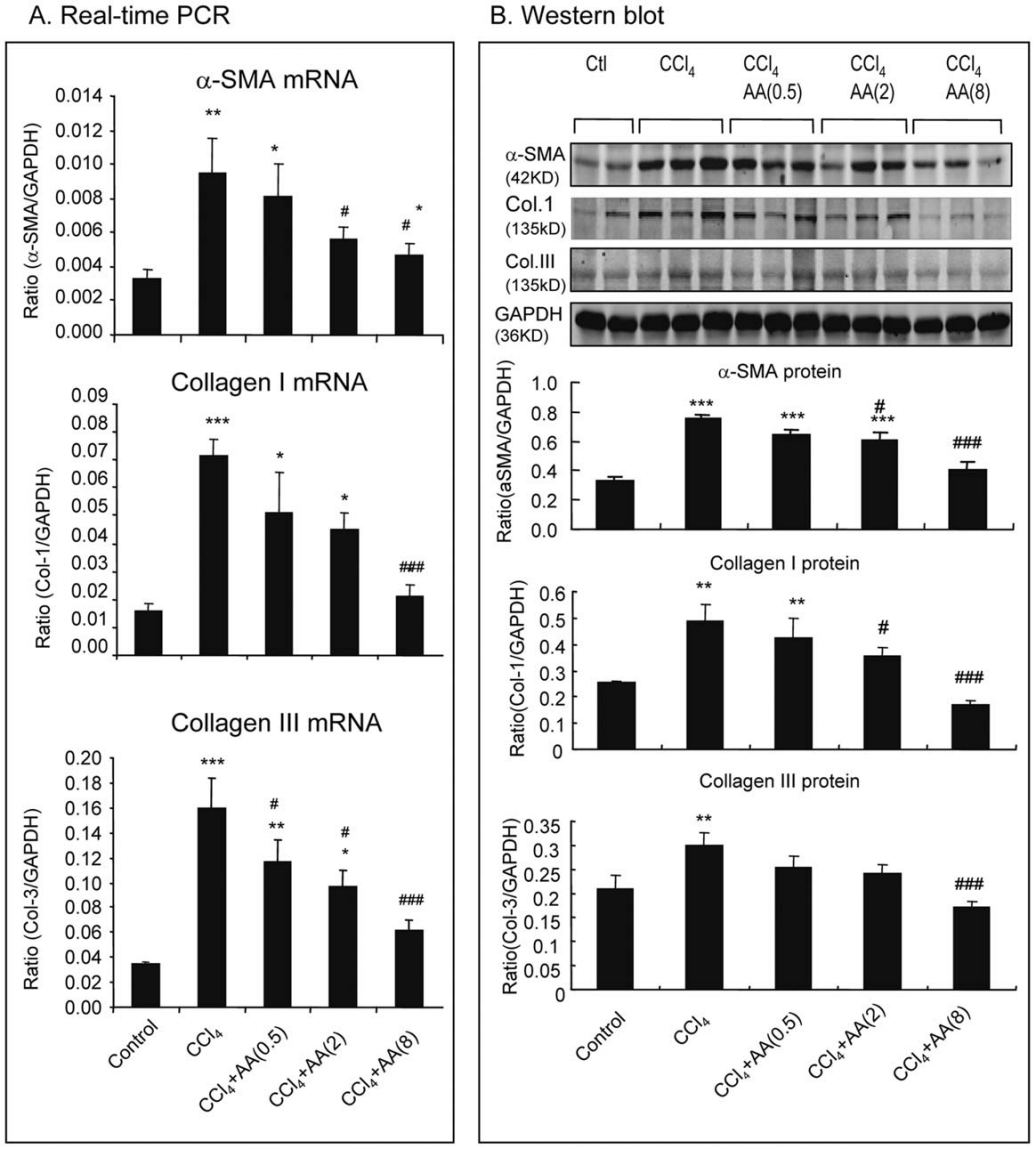
Real-time PCR and Western blot analysis show that AA treatment blocks CCl_4_-induced liver fibrosis in a dosage-dependent manner in rats. **A.** Real-time PCR for a-SMA, collagen type I and III mRNA expression. **B.** Western blot analysis for a-SMA, collagen type I and III protein expression. Data represent mean ± SEM for groups of 8 animals. *p<0.05, **p<0.01, *** p<0.001 versus normal control; ^#^p<0.05, ^###^p<0.001 versus CCl_4_-induced disease control.

The inhibitory effect of AA on liver fibrosis was also demonstrated by at the mRNA levels by real-time PCR. As shown in Figure 4(A), CCl_4_-induced upregulation of a-SMA and collagen type I and III mRNA was significantly attenuated in those treated with AA in a dose-dependent manner. These findings were also evidenced by Western blot analysis (Fig. 4B).

### Asiatic Acid Inhibits TGF-beta1-Induced HSC Activation of Collagen Matrix Expression by HSC-T6 Cells *in Vitro*

**Figure 5:**
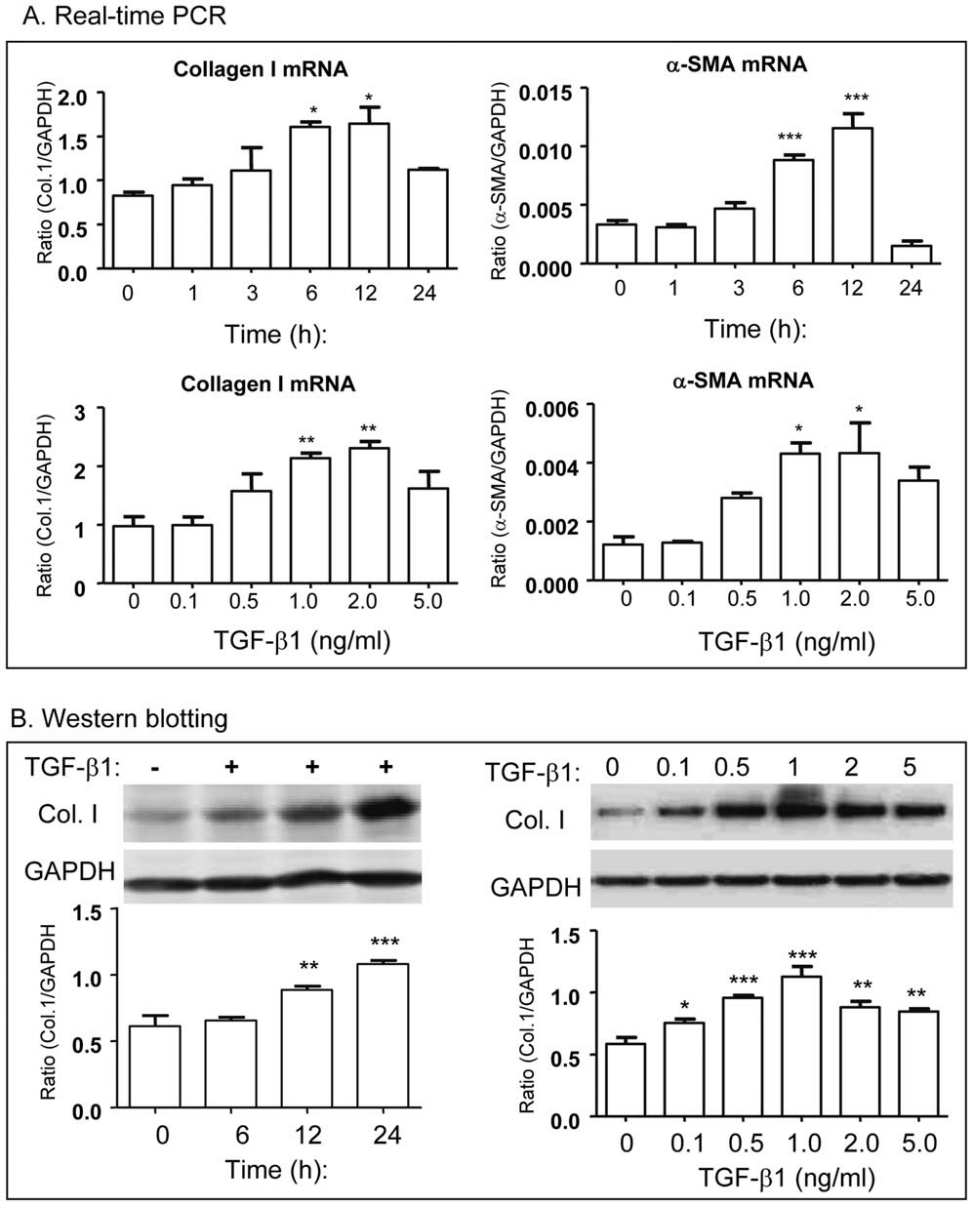
TGF-beta1 induces collagen I and a-SMA expression by HSC-T6 cells *in vitro*. **A.** Real-time PCR show that TGF-beta1 (1 ng/ml) induces collagen I and a-SMA mRNA expression in a time and dosage-dependent manner. **B.** Western blot analysis for a time (TGF-beta1 1 ng/ml) and dosage (24 h)-dependent effects of TGF-beta1 on collagen I expression. Data represent mean ± SEM for at least 3 independent experiments. *p<0.05, **p<0.01, ***p<0.001 versus medium control.

Because TGF-beta1 has been long considered as a key mediator in the pathogenesis of liver fibrosis [1]–[4], we examined if AA is able to inhibit the fibrotic effects of TGF-beta1 on ECM expression in a well-characterized HSC-T6 cells. We first determined an optimal dose of TGF-beta1 in fibrosis response on HSC-T6 cells. As shown in Figure 5, both real-time PCR and Western blot analyses detected that addition of TGF-beta1 induced collagen I and a-SMA mRNA and protein expression in a time- and dosage-dependent manner, being an optimal dose of TGF-beta1 at 1 ng/ml with the peaked time for mRNA expression at 6 h and protein expression at 24 h.

**Figure 6:**
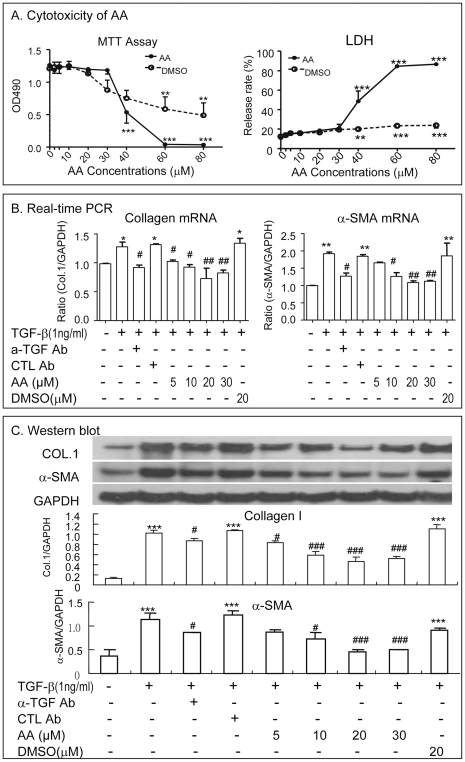
AA inhibits TGF-beta1-induced collagen I and a-SMA expression by HSC-T6 cells in a dosage-dependent manner *in vitro*. **A.** Dose-dependent effects of AA on cytotoxicity of HSC-T6 cells by MTT and LDH release assays. **B and C.** Real-time PCR and Western blot analysis for a dosage-dependent inhibitory effect of AA on collagen type I and a-SMA expression. Data represent mean ± SEM for at least 3 independent experiments. *p<0.05, **p<0.01, ***p<0.001 versus medium control; ^#^p<0.05, ^##^p<0.01, ^###^p<0.001 versus TGF-beta1-treated, isotype control antibody-treated (CTL Ab), or DMSO-treated cells.

We then determined the safe dose of AA without causing cytotoxicity for the *in vitro* study in HCS-T6 cells. As shown in Figure 6(A), AA at doses over 40 micro molar (uM) caused a significant cytotoxicity on HSC-T6 by inhibiting HSC-T6 cell proliferation (MTT assay) and increasing LDH release. In contrast, there was not detectable cytotoxicity when doses of AA were at and below 30 µM. Thus, safe doses of AA (0, 5, 10 20, 30 uM) were used for studying the inhibitory effect of AA on TGF-beta1 (1 ng/ml)-induced HSC activation and ECM production *in vitro*. As shown in Figure 6(B,C), real-time PCR demonstrated that addition of AA significantly inhibited TGF-beta1-inudced collagen I and a-SMA mRNA expression in a dosage-dependent manner, being an optimal dose at 20-30 uM (Fig. 6B). Similar results were also observed at the protein levels as demonstrated by Western blot analysis (Fig. 6C).

### Upregulation of Hepatic Smad7, thereby inhibiting TGF-beta/Smad signaling, Is a Key Mechanism by Which Asiatic Acid Attenuates hepatic fibrosis *in vivo* and *in vitro*

**Figure 7:**
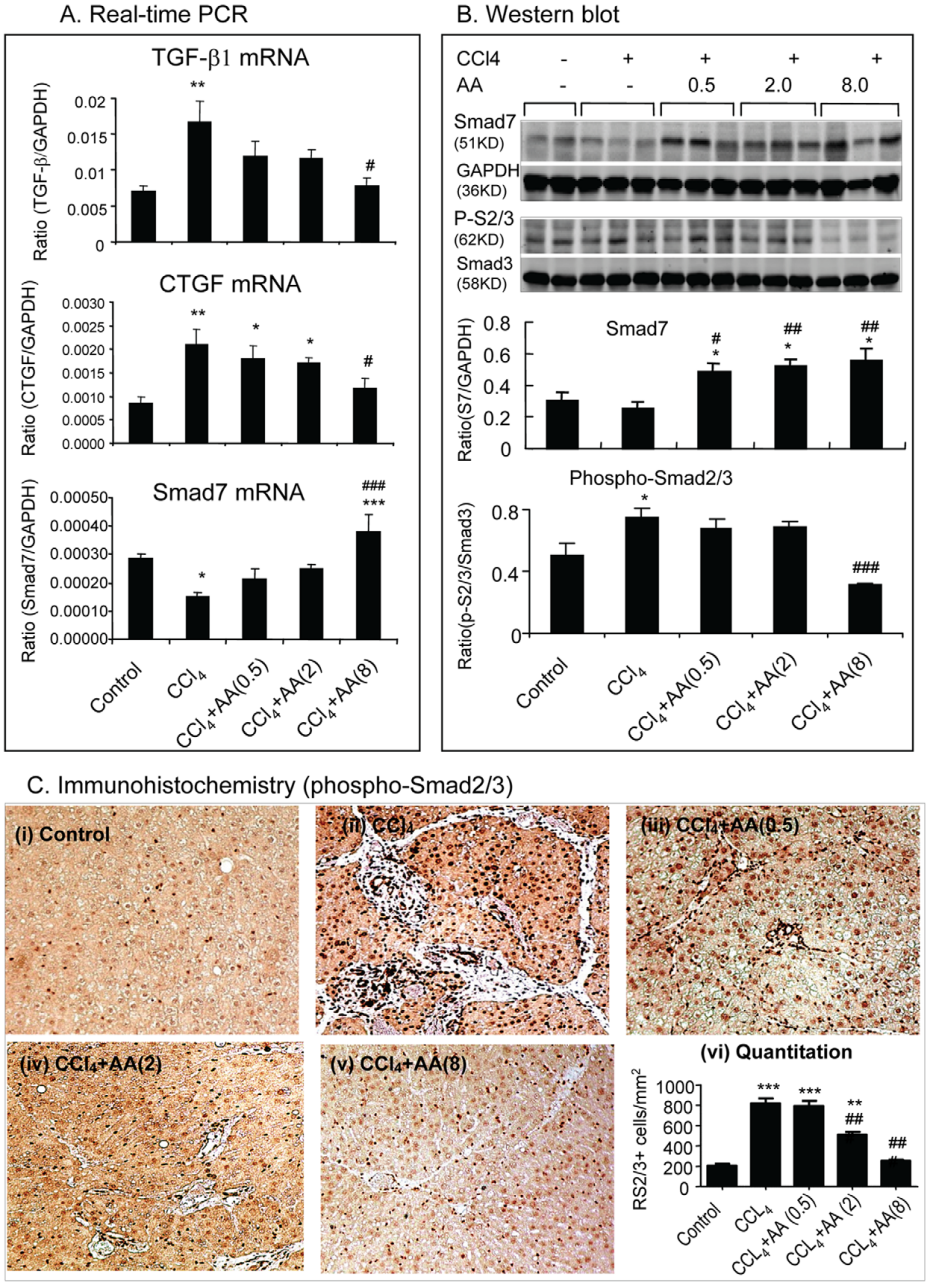
AA treatment upregulates hepatic Smad7, but blocks expression of TGF-beta1 and CTGF and activation of Smad2/3 in CCl_4_-induced liver disease in rats. **A.** Real-time PCR analysis of TGF-beta1, CTGF, and Smad7 mRNA expression. **B**. Western blot analysis for levels of phospho-Smad2/3 and Smad7 protein expression. **C.** Immunohistochemistry for nuclear location of phospho-Smad2/3. Data represent mean ± SEM for groups of 8 animals. *p<0.05, **p<0.01, ***p<0.001 versus normal control; ^#^p<0.05, ^##^p<0.01, ^###^p<0.001 versus CCl_4_-induced disease control. Magnification: ×200 (C).

Since TGF-beta/Smad signaling is a key pathway leading to liver fibrosis [8]–[11], we then investigated the mechanisms by which AA attenuates CCl_4_-induced liver fibrosis by examining the TGF-beta/Smad signaling pathway. As shown in Figure 7, compared to normal control rats, CCl_4_-induced liver fibrosis was associated with a marked upregulation of TGF-beta1 and CTGF mRNA (Fig. 7A), which was associated with a marked activation of Smad2/3 as identified by higher levels of phospho-Smad2/3 and its nuclear translocation (Fig.7B,C), and a fall of hepatic Smad7 (Fig. 7A and B). In contrast, diseased rats treated with AA significantly reduced TGF-beta and CTGF mRNA expression and blocked activation of Smad2/3 in a dosage-dependent manner (Fig. 7). Importantly, the inhibitory effect of AA on TGF-beta/Smad signaling was associated with a marked upregulation of hepatic Smad7 as demonstrated at the mRNA level by real-time PCR and at the protein level by Western blot analysis (Fig. 7 A and B).

**Figure 8:**
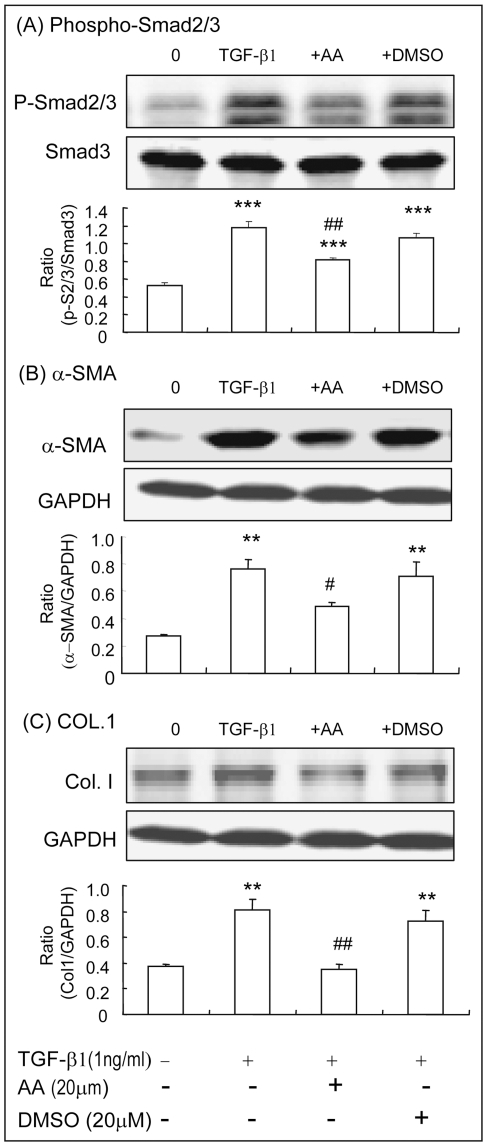
AA blocks TGF-beta1-induced phosphorylation of Smad2/3, a-SMA, and collagen matrix production by HSC-T6 cells *in vitro*. Western blot analysis detects that HSC-T6 cells pretreated with AA (20 uM) for overnight blocks TGF-beta1 (1 ng/ml)-induced Smad2/3 phosphorylation at 30 mins (**A**) and upregulation of α-SMA and collagen I expression at 24 h (**B,C**). Data represent mean ± SEM for at least 3 independent experiments. **p<0.01, ***p<0.001 versus medium control; ^#^p<0.05, ^##^p<0.01 versus TGF-beta1-treated or DMSO-treated cells.

**Figure 9:**
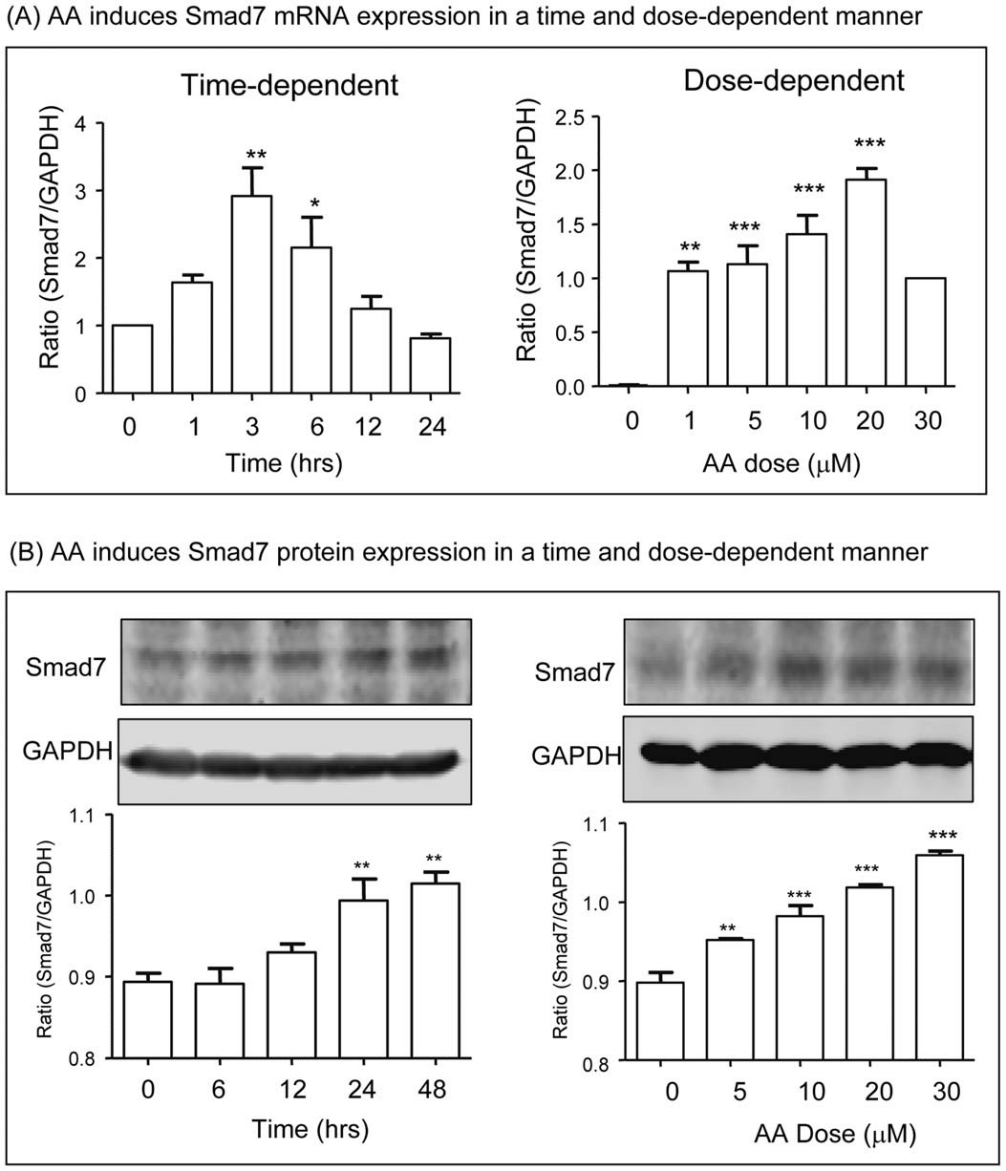
AA induces Smad7 expression by HSC-T6 cells in a time and dosage-dependent manner *in vitro*. **A.** Real-time PCR. **B.** Western blots. Results show that addition of AA induces Smad7 mRNA and protein expression in a time (at a dose of 20 uM) and dosage (3 h for mRNA and 24 h for protein)-dependent manner. Data represent mean ± SEM for at least 3 independent experiments. *p<0.05, **p<0.01, ***p<0.001 versus medium control.

**Figure 10:**
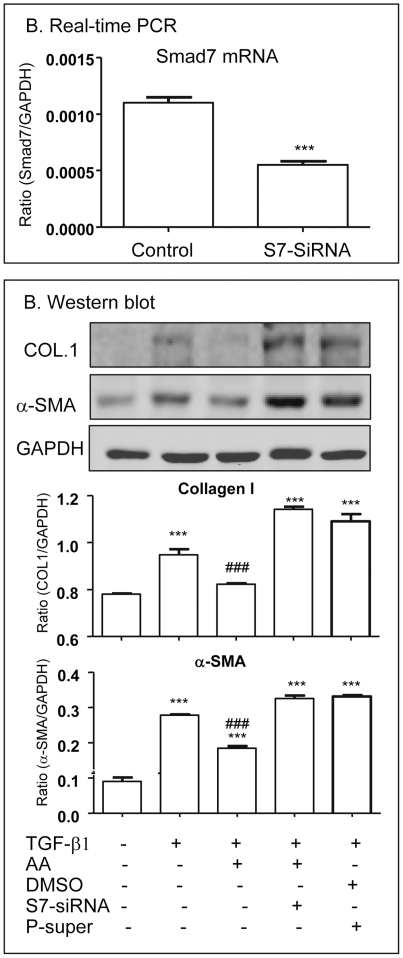
Knockdown of Smad7 from HSC-T6 cells prevents the inhibitory effect of AA on TGF-beta1-induced hepatic fibrosis *in vitro*. **A.** Real-time PCR shows reduction of Smad7 mRNA by siRNA technique. **B.** Western blot analysis detects that knockdown of Smad7 from HSC-T6 cells results in a loss of AA (20 uM)-induced inhibition of TGF-beta1 (1 ng/ml)-mediated collagen I and a-SMA expression at 24 h. Data represent mean ± SEM for at least 3 independent experiments. *p<0.05, **p<0.01, ***p<0.001 versus control; ^###^p<0.001 versus TGF-beta1, DMSO, and control vector (P-super)-treated cells.

The mechanism of AA-induced upregulation of hepatic Smad7 to inhibit CCl_4_-induced liver fibrosis was further investigated in vitro by knocking down Smad7 in HSC-T6 cells. Western blot analysis detected that addition of AA, but not DMSO, was capable of blocking TGF-beta1-induced phosphorylation of Smad2/3 and α-SMA and collagen I expression by HSC-T6 cells (Fig.8). The inhibitory effect of AA in TGF-beta/Smad-mediated hepatic fibrosis was associated with upregulation of Smad7 as demonstrated by the findings that AA alone was able to induce Smad7 mRNA and protein in a time and dosage-dependent manner (Fig. 9 A and B). To further examine the hypothesis that AA induces Smad7 to inhibit TGF-beta1-mediated hepatic fibrosis, Smad7 gene was knocked down from HSC-T6 cells by siRNA technique. As shown in Figure 10, knockdown of Smad7 from HSC-T6 cells was able to prevent the inhibitory effect of AA on TGF-beta1-induced collagen I and a-SMA expression.

## Discussion

Although it is now well accepted that TGF-beta/Smad signaling is a major pathway leading to end-stage liver failure featuring with cirrhosis, treatments for hepatic fibrosis remain non-specific and ineffective. In this study, we reported here that AA, a natural product from *Centella asiatica*, may be a novel therapeutic agent for liver fibrosis. Administration of AA significantly inhibited CCl_4_-induced activation of HSC and liver fibrosis and largely improved liver functional injury in a dosage-dependent manner in rats. In addition, we also found that addition of AA was able to block TGF-beta1-induced HSC activation such as a-SMA+ myofibroblast transition and collagen matrix expression in a rat HSC-T6 cell line. More importantly, upregulation of hepatic Smad7, thereby blocking TGF-beta/Smad signaling, may be the underlying mechanism by which AA attenuated CCl_4_-induced liver fibrosis *in vivo* and TGF-beta1-stimulated HSC activation and ECM production *in vitro*.

In the context of liver fibrosis, TGF-beta1 is a key mediator to activate HSCs to transform into a-SMA+ myofibroblast-like cells, a cell type producing ECM during fibrogenesis [2]–[5]. It has been shown that AA derivatives are able to inhibit ECM production by HSC and keloid fibroblasts by blocking the autocrine effect of TGF-beta1 *in vitro* [32], [33]. The present study added new information that AA was capable of blocking exogenous TGF-beta1-induced myofibroblast transition and collagen I matrix expression by HSC, suggesting that AA may counter-regulate the profibrotic effect of TGF-beta1 in liver fibrosis. This was further confirmed *in vivo* in a rat model of CCl_4_-induced hepatic fibrosis in which treatment with AA significantly attenuated CCl_4_-induced liver fibrosis and functional injury. All these findings demonstrated that AA may be a novel and effective therapeutic agent for hepatofibrosis.

A novel and significant finding in the present study was the identification that AA-induced upregulation of hepatic Smad7, thereby inhibiting TGF-beta/Smad signaling, was a mechanism by which AA inhibits CCl_4_ or TGF-beta1-induced HSC activation and liver fibrosis *in vivo* and *in vitro*. Indeed, activation of TGF-beta/Smad signaling is a key mechanism of liver fibrosis in both experimental and human chronic liver diseases [2]–[4]. The functional importance of TGF-beta/Smad signaling in liver fibrosis has been demonstrated by the finding that disruption of Smad3 protects against dimethylnitrosamine-induced hepatic fibrosis [8]. In contrast, deletion of Smad7, an inhibitor of TGF-beta/Smad signaling, enhances CCl_4_-induced liver damage and fibrosis in mice [9]. In the present study, CCl_4_-induced liver fibrosis was associated with a marked activation of Smad2/3 but a loss of Smad7, suggesting that the imbalance between Smad2/3 and Smad7 signaling could be important in the pathogenesis of liver fibrosis. This is confirmed by the recent studies that overexpression of Smad7 in the liver attenuates TGF-beta/Smad signaling and protects against HSC activation and liver fibrogenesis in CCl_4_-induced mouse and rat models [10], [11]. Although the mechanisms of TGF-beta/Smad-mediated liver fibrosis are well understood, the development of therapeutic drugs directly targeting this pathway remains unexplored. The present study identified that treatment with AA was able to induce hepatic Smad7, thereby blocking TGF-beta/Smad signaling and fibrosis in a rat model of CCl_4_-induced liver fibrosis and in TGF-beta1-activated HSC *in vitro*. These results suggest that induction of Smad7, thereby restoring the balance of TGF-beta/Smad signaling, may be a central mechanism by which AA inhibits liver fibrosis *in vivo* and *in vitro*. This was supported by the finding that knockdown of Smad7 was able to protect against HSC from TGF-beta1-induced activation and fibrosis *in vitro*.

In summary, the present study demonstrates that AA may be a novel therapeutic agent for liver fibrosis. Induction of hepatic Smad7, thereby inhibiting activation of TGF-beta/Smad signaling, may be an underlying mechanism by which AA protects against chronic liver disease associated with fibrosis.
